# Small RNA Differential Expression Analysis Reveals miRNAs Involved in Dormancy Progression in Sweet Cherry Floral Buds

**DOI:** 10.3390/plants11182396

**Published:** 2022-09-14

**Authors:** Esteban Soto, Evelyn Sanchez, Carlos Nuñez, Christian Montes, Karin Rothkegel, Paola Andrade, Humberto Prieto, Andrea Miyasaka Almeida

**Affiliations:** 1 Instituto de Investigaciones Agropecuarias, INIA La Platina, La Pintana 8831314, Chile; 2 Escuela de Bioquímica, Facultad de Ciencias, Universidad Andrés Bello, Santiago 8370251, Chile; 3 Institute of Crop Science and Resource Conservation (INRES), Horticultural Sciences, University of Bonn, 53121 Bonn, Germany; 4 Centro de Genómica y Bioinformática, Facultad de Ciencias, Ingeniería y Tecnología, Universidad Mayor, Camino La Pirámide 5750, Huechuraba 8580745, Chile; 5 Centro de Biotecnología Vegetal, Facultad de Ciencias, Universidad Andrés Bello, Santiago 8370146, Chile; 6 Escuela de Agronomía, Facultad de Ciencias, Ingeniería y Tecnología, Universidad Mayor, Huechuraba 8580745, Chile

**Keywords:** *Prunus avium*, miRNA, chilling requirement, flowering

## Abstract

In sweet cherry (*Prunus avium*), as in other temperate woody perennials, bud dormancy allows for survival in adverse environmental conditions during winter. During this process, environmental signals such as short days and/or low temperatures trigger internal signals that enable buds to become tolerant to the cold. The process involves tracking chilling units up to chilling the requirement fulfillment to resume growth, a transition involving transcriptional regulation, metabolic signaling, and epigenetic-related regulatory events. Massive sequencing of small RNAs was performed to identify miRNAs involved in sweet cherry dormancy by comparing their expression in field (regular seasonal) and controlled non-stop (continuous) chilling conditions. miRNAs highlighted by sequencing were validated using specific stem-loop PCR quantification, confirming expression patterns for known miRNAs such as miR156e, miR166c, miR172d, miR391, miR482c, and miR535b, as well as for newly proposed miRNAs. In silico prediction of the target genes was used to construct miRNA/target gene nodes. In particular, the involvement of the sweet cherry version for the miR156/*SQUAMOSA PROMOTER-BINDING-LIKE PROTEIN* genes whose expression was opposite in the two conditions suggests their involvement on dormancy regulation in sweet cherry. miRNA levels indicate that the regulation of stress-related genes and hormone synthesis modulates the expression of calcium metabolism and cell development-associated genes. Understanding the regulatory networks involved in sweet cherry dormancy, particularly in the context of miRNA involvement, represents the first step in the development of new agricultural strategies that may help overcome the increasing challenges presented by global climate change.

## 1. Introduction

Woody perennial plants, including the deciduous fruit trees of temperate climates, such as *Prunus avium* (sweet cherry tree), survive adverse winter conditions through a process called dormancy. During summer, these trees generate protective structures called buds, which contain meristematic tissue responsible for the development of flowers and leaves [1]. In autumn, trees stop vegetative growth and initiate dormancy, and the leaves fall spontaneously until the tree is completely defoliated. Lang (1987) proposed that dormancy could be classified according to the physiological state into paradormancy, endodormancy, and ecodormancy [2]. Paradormancy refers to growth inhibition due to apical dominance, endodormancy refers to endogenous inhibition within the meristem, and ecodormancy refers to growth inhibition due to low temperatures [2]. In endodormancy, the trees must complete a chilling requirement (CR) during winter, and afterwards a heat requirement in early spring (ecodormancy), to resume both vegetative and reproductive growth during spring and summer [2,3]. Because the fulfillment of a CR is specific for each variety or genotype [4,5], the climatic conditions of a location must match the CR of the tree species and cultivars. Symptoms of insufficient CR include incomplete breaking of dormancy, delayed bloom, heterogeneous fruit maturation, low yields, and poor fruit quality [5,6]. However, in locations where the chill exceeds the CR of trees, they may flower too early, leading to an elevated risk of spring frost [7]. Previous studies on dormancy have focused on genetic control, showing that CR is a major determinant of the flowering date in peach (*P. persica*), sweet cherry, and almond (*P. dulcis*) [8,9,10]. However, it is difficult to determine whether CR has been fulfilled, because buds remain on the tree without visible morphological changes during dormancy. The transition from dormancy to budburst involves the integration of transcriptional regulation, metabolic signaling, and epigenetic regulatory events [11,12,13]. In *Prunus* spp., including sweet cherry, this transition involves histone modification, RNA interference, and the production of small RNAs (sRNAs) involved in gene silencing [13,14]. Examples of these changes have been observed in the promoters and introns of some members of the *DORMANCY-ASSOCIATED MADS-BOX* gene family [11,12,14], an ortholog of the *Arabidopsis thaliana* flowering factor SHORT VEGETATIVE PHASE (SVP) [11].

sRNAs are short non-coding RNAs ranging from 20- to 24-nucleotides (nt) in length, many of which have been associated with environmental plant responses, involving events linked to genome reprogramming and plant development [13]. A particular sRNA subset, named microRNAs (miRNAs; ranging between 21- and 22-nt in length), was first described as a regulator of gene expression in *Caenorabditis elegans* in the 1990s [15]. The biosynthesis of miRNAs in plants begins with the transcription of the miRNA gene by RNA POLYMERASE II. The resulting transcript, called pri-miRNA, is 5′ capped, 3′-polyadenylated, and/or spliced [16]. The pri-miRNA then folds through the interaction of complementary internal sequences to form a hairpin (stem loop). A protein with ribonuclease activity, DICER-LIKE 1, processes this pre-miRNA to form one or more duplexes of miRNA that are exported to the cytoplasm [16,17]. The mature miRNA is then integrated into an RNA-induced silencing complex composed of the ARGONAUTE 1 protein and 21-nt miRNA. This RNA-protein complex recognizes its target mRNA and can either directly degrade it or inhibit its translation [16]. Trans-acting siRNAs (tasiRNAs) are 21-nt siRNAs generated by the action of miRNAs that cleave tasiRNA-generating (TAS) gene transcripts. The cleavage products from these events are useful as a substrate for the synthesis of double-stranded RNAs that are processed, step-by-step, in a process known as ‘phasing’, to form the tasiRNAs populations. These molecules have been described as regulating the expression (by cleaving or repressing the translation) of target mRNAs involved in the vegetative phase transition of the Arabidopsis TAS3 locus [18].

Today, miRNAs are known to play crucial roles in plant–environment interactions [19]. The number of known miRNA genes has rapidly increased because of microarray and next-generation sequencing (NGS) technologies. Differential expression of miRNAs during dormancy and chilling accumulation has been observed in fruit trees such as peaches [20], pears (*Pyrus pyrifolia*) [21,22], almonds [23], and apples [24]. In the case of pears, two independent groups reviewed the expression of miRNAs during dormancy and found that miR156 was upregulated during chill accumulation [21,22]. miR156 binds directly to the 3’-UTR of genes of the *SQUAMOSA PROMOTER-BINDING-LIKE PROTEIN (SPL)* gene family [25,26]. miR156 regulates the expression of miR172 via *SPL9*, which redundantly with *SPL10* directly promotes the transcription of miR172b in *Arabidopsis* [27]. SQUAMOSA PROMOTER-BINDING-LIKE PROTEIN is a transcription factor that binds directly to the promoters of *FLOWERING LOCUS T (FT*), *SUPRESSOR OF OVEREXPRESSION OF CONSTANS 1* (*SOC1*), *FRUITFULL* (*FUL*), *LEAFY* (*LFY*), and *APETALA 1* (*AP1*) genes to promote flowering [28]. The targets of miR172 are *APETALA2* (*AP2*), *SCHLAFMUTZE* (*SMZ*), *SCHNARCHZAPFEN* (*SNZ*), *TARGET OF EAT1* (*TOE1*), *TOE2,* and *TOE3* genes, which repress flowering [28]. miR172 levels were found to be downregulated by the direct binding of SVP cold-sensitive protein to the CArG motifs in the promoter of the *miR172* gene in *Arabidopsis* [29,30,31].

The molecular events occurring in flower buds during dormancy in *Prunus avium* are less clearly understood, and determining the extent of the involvement of miRNAs in these events could significantly contribute to the development of improved agricultural practices for tree cultivation in the context of climate change. This study aimed to identify sweet cherry miRNAs associated with dormancy in floral buds obtained under field chilling (FC) and non-stop chilling (NC) conditions. Known and new miRNAs were studied using sRNA NGS followed by specific stem-loop validation, and the comparison of miRNA expression in the two conditions allowed for the in-silico identification of highlighting events taking place during the process. In addition, the involvement of the miR156 node, which included *PavSPL2*, *6*, and *13* genes as putative targets, revealed that the expression patterns of miR156e and *SPL13* were opposite in the two conditions, indicating the involvement of this post-transcriptional regulation system in the process.

## 2. Results

### 2.1. Identification and Annotation of miRNAs during Dormancy in Prunus avium L. var. Bing by Next-Generation Sequencing

**Figure 1:**
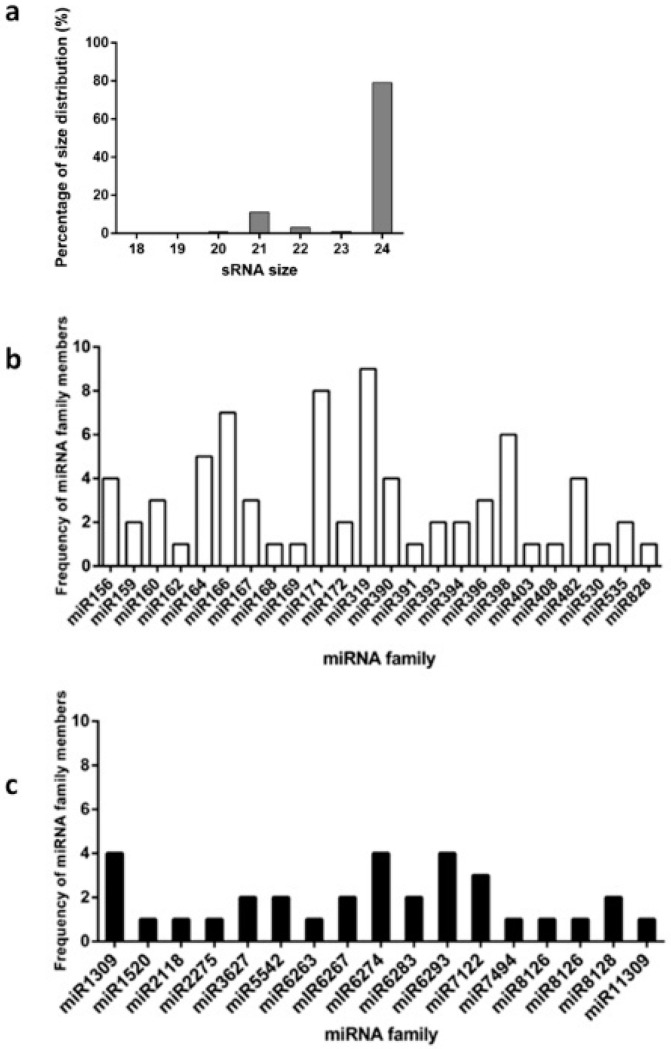
Small RNA next-generation sequencing results and miRNA identification in sweet cherry floral buds during chilling accumulation. Bing floral buds were used as the sRNA source for progressive chilling accumulation under field chilling (natural cold accumulation in the field) conditions. (**a**) Percentage of size distribution of total sRNAs identified with lengths of between 18- and 24-nt. Distribution of highly conserved (**b**) and conserved (**c**) miRNA families.

Small RNA samples from floral buds obtained from the FC conditions were subjected to NGS analysis. Based on the results of chilling requirement determination experiments, floral buds from three dormancy stages were considered for sequencing (Table 1): T0 floral buds from branches before commencement of the chill accumulation (paradormancy), T1 floral buds during chill accumulation (endodormancy), and T2 floral buds after the fulfillment of chilling requirement (CR) (ecodormancy). A total of 10 sequencing libraries (four for T0, three for T1, and three for T2) (Supplementary Table S1) generated 16.9 million total reads and over 8.8 million cleaned reads per library on average. Figure 1a shows the size distribution of these cleaned sRNA reads, ranging between 18- and 24-nt in length, with 21- and 24-nt molecules showing the highest accumulation (11% and 79%, respectively). To identify miRNAs in this dataset, molecules ranging between 19- 22-nt were compared to those in available databases, and a total of 172 sequences were consequently selected (Supplementary Tables S2 and S3). Seventy-four highly conserved miRNAs were found to be distributed in 25 families, of which miR319 had the largest number of members (nine members; Figure 1b). Similarly, 32 conserved miRNAs were found to be distributed in 22 families, of which the miR6274 and miR6293 families had a relatively higher number of members (Figure 1c). In addition, 76 miRNAs that were predicted to have a stem-loop structure but did not match a mature miRNA sequence reported in the miRBase database were annotated as newly identified *P. avium* miRNAs (Supplementary Table S4).

### 2.2. Expression Profiles of miRNAs during Dormancy in Prunus avium L. var. Bing

**Figure 2:**
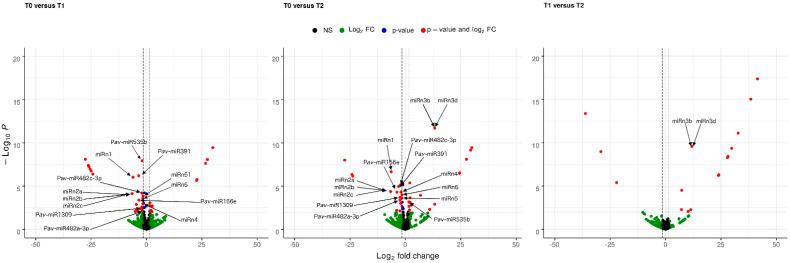
Identification of differentially expressed *Prunus avium* miRNA-like molecules. Pairwise comparisons of the differentially expressed miRNAs between three different cold chilling hours’ (CH) accumulation of field chilling (FC) conditions, denoted by T0 (paradormancy, 0 CH), T1 (endodormancy, 853 CH), and T2 (ecodormancy, 909 CH). Comparisons are indicated on the graphs. Red dots indicate miRNA-like molecules that show log fold change ≥1.5 (*x*-axis), as well as high statistical significance (*p*-value ≤ 0.01, *y*-axis). Blue dots indicate miRNA-like molecules that show log fold change ≤1.5 (*x*-axis), despite the high statistical significance (*p*-value ≤ 0.01, *y*-axis). Green dots indicate miRNA-like molecules that log fold change ≥1.5 (*x*-axis) but lower statistical significance (*p*-value ≥ 0.01, *y*-axis).

**Figure 3:**
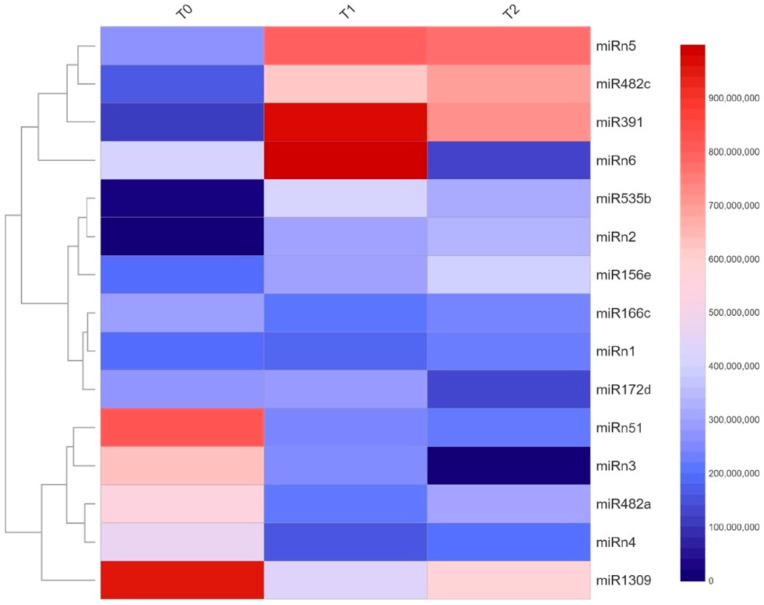
Expression profiles of miRNAs in samples of sweet cherry flower buds during dormancy. Differential expression of miRNAs among the three experimentally defined dormancy stages, namely T0 (0 CH), T1 (853 CH), and T2 (909 CH) using normalized average and hierarchically clustered reads. Expression scale is shown.

Differential expression of miRNAs between the three experimentally defined dormancy stages (T0, T1, and T2) was observed using normalized (as transcript per million values, TPM) and hierarchically clustered (using DeSeq2-Bioconductor) reads. In Figure 2, meaningful changes found in these datasets (red dots) correspond to miRNA-like molecules showing a large-scale fold change ≥ 2, as well as a high statistical significance (*p*-value ≤ 0.05). Thus, 15 miRNAs, namely miR156a, miR166c, miR172d, miR391, miR482a, miR482c, miR535b, miR1309, miRn1, miRn2, miRn3, miRn4, miRn5, miRn6, and miRn51, with fold-changes > 1.5 (Figure 3), were selected for validation.

**Figure 4:**
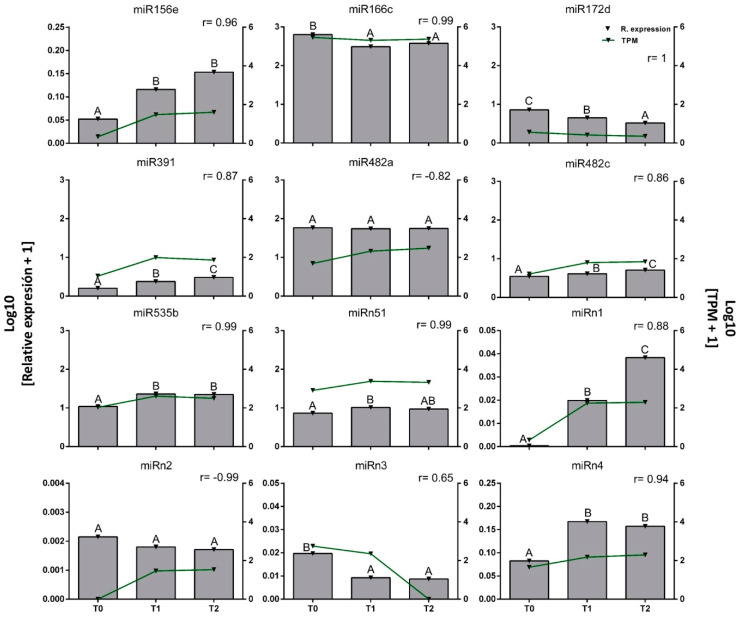
Validation of miRNA expression. The samples from T0 (0 CH), T1 (853 CH), and T2 (909 CH) stages were compared between NGS (lines) and qRT-PCR (bars) determinations. Means with different letters among stages show high statistical significance (*p*-value ≤ 0.05) of three technical replicates. The value of r corresponds to the Pearson’s correlation between the relative expression for qRT-PCR and normalized reads from NGS.

Experimental validation of these NGS-derived data was carried out by quantitative reverse transcriptase PCR (qRT-PCR) relative quantification based on the “precursor_993” molecule, which showed stable expression during dormancy (Figure 4). A measure of linear correlation between the two sets of data obtained from NGS and qRT-PCR was observed based on Pearson’s correlation coefficient (r). A perfect positive correlation (r ≈ 1) allowed the identification of miR156e, miR391, miR482c, miR535b, miRn1, and miRn4, which showed increased expression from T0 to T2. In contrast, miR166c, miR172d, and miRn3 levels decreased from T0 to T2. The identities of these miRNAs were confirmed by Sanger sequencing (Supplementary Figure S1). In contrast, miR482a, miRn2, and miRn51 (with r values of −0.82, −0.99, and 0.99, respectively), showed uneven trends between methods and were therefore not further investigated in this study.

### 2.3. Effect of Environmental Fluctuations on the Levels of miR156e, miR172d, miR482c, miRn1, miRn2, and miRn3

**Figure 5:**
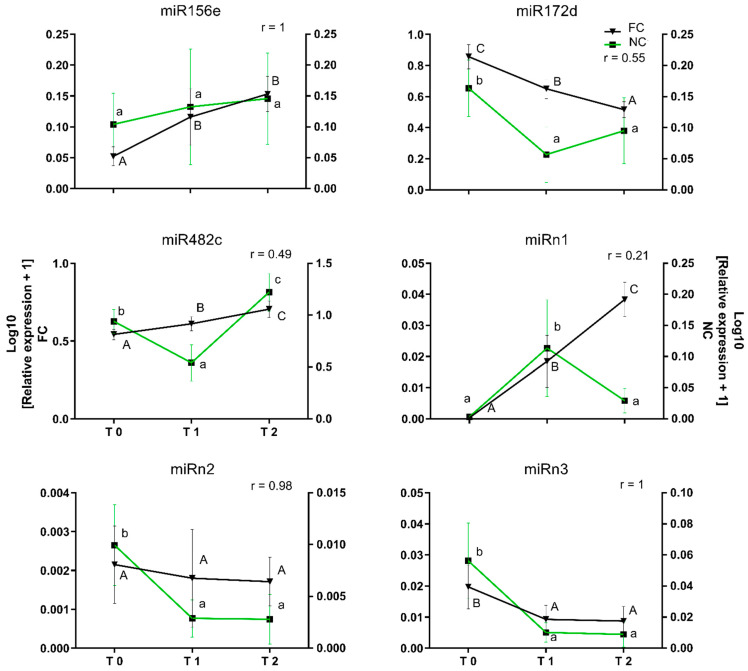
Comparison of the relative expression of miRNAs in floral buds from field chilling (FC) and non-stop chilling (NC) conditions. Floral buds were obtained from trees subjected to natural cold accumulation in the field (FC) and from sticks subjected to continuous chilling under cold storage (NC) conditions. sRNAs were isolated, and expression levels of miRNAs were determined by qRT-PCR. Means with different letters among stages are significantly different (*p*-value ≤ 0.05). Pearson’s correlation (r) was determined between the relative expression levels in both seasons. Black line shows FC relative expression and green line shows NC relative expression.

The effect of environmental variations occurring under FC, compared to controlled uniform conditions in NC, was analyzed by selecting six validated miRNAs, three with a known biological relevance (i.e., miR156e, miR172d, and miR482c), and three from those identified as novel and showing interesting expression patterns (miRn1, miRn2, and miRn3). miRNA analysis for NC was performed in the same way as previously described for FC using qRT-PCR. In Figure 5, the qRT-PCR data obtained from FC (black lines) were complemented with the expression patterns of the miRNAs obtained by NC. miR156e, miRn2, and miRn3 showed identical expression patterns under both conditions, with r values of ~1. In contrast, mir172d, miR482c, and miRn1 showed differences between the conditions, with r values of 0.55, 0.49, and 0.21, respectively.

### 2.4. Identification of the Target Genes of miRNAs in Prunus avium L. var. Bing

Because miRNAs regulate gene expression by sequence recognition, an in silico search of the target genes of sweet cherry miRNAs was performed with the psRNAtarget tool (Supplementary Tables S5–S7), using the transcriptome *of Prunus mume* as a reference [32]. Based on the results, highly relevant molecules and their candidate target genes associated with dormancy are indicated in Table 2, which in summary corresponded to the following miRNA/target gene couples: miR156e/*SQUAMOSA PROMOTER-BINDING-LIKE PROTEIN (SPL)*, miR166c/*AP2-LIKE*, miR172d/*ETHYLENE-RESPONSIVE TRANSCRIPTION FACTOR* *(AP2/ERF)*, miR391/*PROFILIN (PFN)*, miR482a/*DISEASE RESISTANCE PROTEIN AT4G27190-LIKE*, miR482c/*PUTATIVE DISEASE RESISTANCE RPP13-LIKE PROTEIN 1 (RPPL1)*, miR535b/*PERIODIC TRYPTOPHAN PROTEIN 2 HOMOLOG (PWP2)*, miR1309/*WD REPEAT-CONTAINING PROTEIN 7 (WDR7)*, miRn1/*PUTATIVE RECEPTOR PROTEIN KINASE ZMPK1 (PK1)*, miRn3/*NINJA-FAMILY PROTEIN (AFP3)*, miRn4/*ARABINOSYLTRANSFERASE RRA3-LIKE (RRA3)*, miRn5/*PSBP DOMAIN-CONTAINING PROTEIN 7* *(PPD7)*, miRn6/*PENTATRICOPEPTIDE REPEAT-CONTAINING PROTEIN AT1G32415*, *mitochondrial (PCMP-E56),* and miRn51/*METAL TRANSPORTER NRAMP5-LIKE (NRAMP5)*.

### 2.5. Functional Validation of miR156 and Its Target Genes SPL2, SPL6, and SPL13

**Figure 6:**
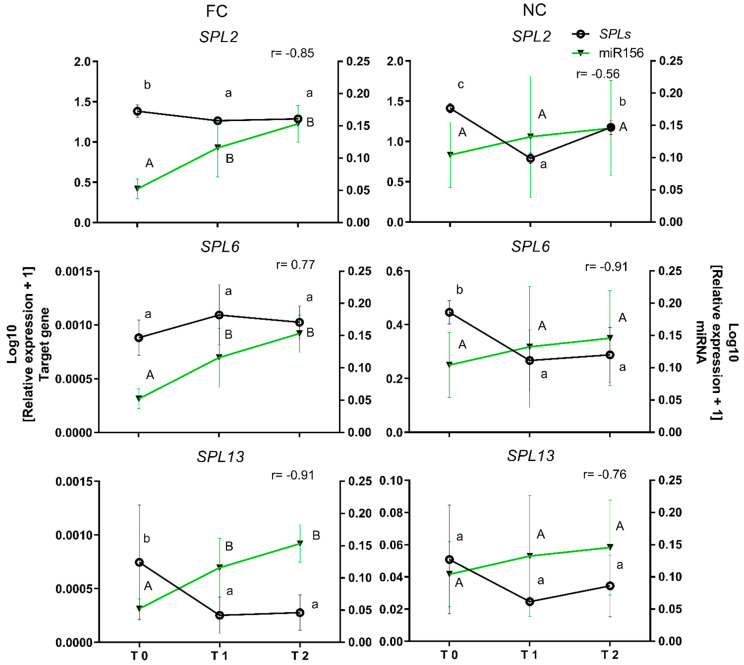
Comparison of miR156 relative expression and its predicted SPL target genes under field chilling (FC) and non-stop chilling (NC) conditions. Floral buds were obtained from trees subjected to natural cold accumulation in the field (FC) and from sticks subjected to continuous chilling under cold storage (NC) conditions. Expression levels of SPL genes and miR156 were determined by qRT-PCR in both experimental conditions. Means with different letters among stages are significantly different (*p* ≤ 0.05). The gene expression levels in FC and NC conditions were compared with miR156 expression levels. Pearson correlation (r) was determined between the relative expression of the SPL gene miR156 in both seasons. Black line, relative expression SPL genes. Green line, relative expression of miR156.

The expected effect of miRNA accumulation is downregulation target gene expression, which can be achieved by miRNA-induced mRNA degradation or translation blockage. In the former case, an inverse correlation should be observed between miRNA and target gene mRNA levels. This possibility was tested for miR156e and its potential targets *PavSPL2*, *PavSPL6,* and *PavSPL13*. Target mRNA levels were compared in flower buds of dormancy states T0, T1, and T2 under both experimental conditions (FC and NC). As illustrated in Figure 6, miR156e expression, which is shown as a green line, showed interesting correlations with each target gene. Based on Pearson’s coefficient, the expression of the *PavSPL2* gene showed the expected inverse correlation with miR156e expression with an r value of −0.85 in FC conditions. However, this was not observed under NC conditions, where the r value was −0.56. Though *PavSPL6* mRNA expression did not show an inverse correlation with the expression of miR156e under FC conditions (r = 0.77), this was not the case under NC conditions, where the r value was −0.91. Finally, as expected, the expression of the *PavSPL13* gene showed an inverse correlation with that of miR156e under both FC and NC conditions, with r values of −0.91 and −0.76, respectively.

## 3. Discussion

### 3.1. CR Determination of Prunus avium L. var. Bing

Varietal CR in sweet cherry is one of the key elements determining adaptation to climatic conditions in the growing area, and its fulfillment leads to adequate flower production and fruit set. The Bing variety originated in Oregon, United States [33]. We determined CR fulfillment for Bing when at least 50% of the buds were in the BBCH 53 stage [1] and based on the Chilling Hours (CH) model under FC (909 CH) and NC (916 CH) conditions (Table 1). The similarity in these values suggests that temperature fluctuations occurring during FC did not have a major influence on CR under our experimental conditions, and that these genetic aspects can strongly determine this element [10]. An in-depth transcriptomic analysis covering all stages of flower buds in three sweet cherry varieties has previously revealed that the expression patterns were very similar, even if the samples were collected on different dates [34]. Increasing evidence indicates the involvement of conserved molecular genetic pathways in the control of dormancy stages in *Prunus* spp., including sweet cherries [4,8,10,34].

In general, it is well known that plants respond to changes in temperature by finely regulating the expression of specific genes [35], and data from *Prunus* spp. such as *P. avium* and *P. persica* indicate that dormancy release depends on the relationship between chilling and heat requirements, as evident by the concurrence of common pathways dependent on changes in temperature [8,10]. Indeed, the relevance of warm temperatures in the coordination of floral bud burst once CR is fulfilled has recently been associated with extensive epigenetic modification of the *DORMANCY-ASSOCIATED MADS-BOX* (*DAM*) *locus* in peach [35], demonstrating that floral buds do not proceed to flowering without exposure to warm temperatures.

### 3.2. Identification of Dormancy Sensitive microRNAs in Prunus avium L. var. Bing by Massive Sequencing

Next-generation massive sequencing has improved the ability to analyze regulatory sRNAs. For instance, genome-wide analyses have already enabled the study of sRNA populations during dormancy in *Malus domestica*, *Pyrus pyrifolia*, *P. persica*, and *Citrus sinensis* (orange) [22,24,36,37]. Consequently, miRNAs involved in chilling-induced dormancy release have already been identified and characterized in pear and apple [21,22,24]. In these studies, sRNA populations ranging between 18- and 24-nt in length were found to be mostly composed of 24- and 21-nt molecules. In Bing, for which we defined three dormancy stages, 79% and 11% of the selected reads were 24- and 21-nt in length, respectively. In addition, we selected molecules ranging between 19- and 22-nt for the identification of miRNAs. This range was used in previous studies on almond and peach, establishing 21 nt-long sRNAs as the most abundant molecules, as also found in our study [23,37]. Homologous sequences were identified in miRBase, and the corresponding names were used to recall the corresponding sweet cherry mature miRNAs (Figure 3; Supplementary Tables S2–S4).

### 3.3. Identification and Characterization of microRNAs and Target Genes during Dormancy in Prunus avium L. var. Bing

Expression analysis was then performed to determine the times of change (log2 fold change) and thus select the miRNAs with the highest differential expression. miRNAs with a change rate greater than 1.5 times during dormancy were selected. This is a strict criterion compared to that commonly used in scientific publications, in which miRNAs are selected with a log2 fold change of up to 0.5 [21,23]. A stricter criterion has the advantage of limiting the results to miRNAs with significant changes in expression.

MicroRNA expression patterns deduced from NGS studies were validated by qRT-PCR, a technique described as having higher resolution in miRNA studies, capable of detecting miRNAs from as little as 20 pg of plant tissue total RNA [38]. We found that 10 of 12 miRNAs validated by qRT-PCR showed a high statistical significance (*p* ≤ 0.05) at some time point. It is important to note that NGS-derived results (expressed in TPM) differ from those obtained by qRT-PCR (expressed according to a reference control); therefore, the abundance of each miRNA is not directly comparable between the techniques, and only their trend over time can be compared. We observed a positive Pearson’s correlation coefficient (r ≈ 1) between the two datasets (obtained from NGS and qRT-PCR) in 9 out of 12 miRNAs, namely miR156e, miR391, miR482c, miR535b, miRn1, miRn4, miR166c, miR172d, and miRn3. In contrast, miR482a, miRn2, and miRn51 showed low correlation between the methods.

The target genes of the miRNAs with a fold change greater than 1.5 times (in TPM) are summarized in the following paragraphs, the biological roles of these miRNA molecules during dormancy are summarized in Table 2, and some relevant deduced pathways are proposed in Supplementary Figure S2.

The target gene for miR156 is *SQUAMOSA PROMOTER-BINDING-LIKE PROTEIN (SPL)* mRNA. miR156 transcriptionally represses *SPL* genes (*SPL2*, *3*, *4*, *5*, *6*, *9*, *10*, *11*, *13,* and *15*) by directly binding to the 3-′ UTR region in *Arabidopsis* [25,26]. SPL binds directly to the promoters of *FLOWERING LOCUS T (FT)*, *SUPPRESSOR OF OVEREXPRESSION OF CONSTANS 1 (SOC1)*, *FRUITFULL (FUL)*, *LEAFY (LFY)*, and *APETALA 1 (AP1)* to promote flowering [26]. In *Arabis alphina*, an increase in miR156 levels was observed during endodormancy, indicating that miR156 expression is influenced by low temperature [39]. Moreover, it has been observed in the leaves of *Solanum commersonii* (a wild potato species) and *Oryza sativa* (rice) that the expression of miR156 increases during cold stress, and that the overexpression of miR156 confers tolerance to these conditions [40,41]. In our study, we observed an increase in the expression of miR156e during dormancy in the sweet cherry, which was similar to previously published studies in pears [21,22].

The target of miR166 is the homeobox-leucine zipper protein *ATHB15* mRNA. Since the 2000s, miR166 has been predicted to transcriptionally repress the homeobox-leucine zipper protein *ATHB15* gene by binding to its mRNA [42]. The overexpression of the *MIR166a* gene in *Arabidopsis* [43] drastically reduced *ATHB15* transcription, causing severe alteration of the vascular system with expanded xylem tissue and interfascicular region [43]. The miR166 complementary sequence is highly conserved in the mRNA of the *ATHB15* gene in several plant species [42]. Therefore, it is likely that miR166-mediated *ATHB15* repression is conserved across all vascular plants as well [44].

In sweet cherries, genes associated with the response to abscisic acid (ABA), such as *ATHB7*, are highly expressed during endodormancy [34]. According to our results, miR166c was downregulated towards dormancy release, which was similar to that which was observed in sRNA libraries of pear flower buds during endodormancy and ecodormancy [22].

The targets of miR172 are *APETALA2 (AP2)*, *SCHLAFMUTZE (SMZ)*, *SCHNARCHZAPFEN (SNZ)*, *TARGET OF EAT1 (TOE1)*, *TOE2,* and *TOE3* [28]. In *Arabidopsis*, overexpression of *AP2* causes a delay in the transition to adulthood [45], whereas repression of *AP2* by miR172 has the opposite effect, releasing flowering [45]. In addition, deletion of the miR172 binding site in the *AP2* gene in peaches causes the double-flower trait [46,47]. In contrast, the *SHORT VEGETATIVE PHASE (SVP)* gene, a *MADS* box transcription factor, is a critical inhibitor of flowering that directly represses the transcription of the *MIR172* gene. SVP binds directly to CArG motifs in the *MIR172* promoter in *Arabidopsis* [19,31]. In our study, the expression of miR172d in sweet cherry was found to be downregulated during dormancy, indicating that *AP2* expression increases flowering. In contrast, under NC conditions, miR172 expression increased when CR was fulfilled (T2 stage). Similar results have been reported for pears [37,38]. This could be explained by the regulation of the *MIR172* promoter by SVP. These results support the hypothesis that the miR172/AP2 regulatory axis plays an important role in promoting temperature-dependent flowering in temperate fruit trees.

According to our predictions, the target of miR391 is the *PROFILIN* gene. PROFILIN is an actin-binding protein involved in the assembly of the actin polymer [48]. However, previous studies indicate that the *ARF16* and *ARF17* genes, which are involved in the regulation of root growth, are the targets of miR391 [49]. Recently, transgenic overexpression of miR391 in *Arabidopsis* demonstrated post-transcriptional silencing of the *ACA-10* gene by this miRNA [50]. ACA-10 codes for a calcium-transporting pump (*AtACA10*) involved in plant development, immunity, and response to the environment. *Arabidopsis* ACA10 mutants exhibit a compact inflorescence phenotype. So far, there is no evidence of the role of miR391 during dormancy; however, in our study, the expression was significantly upregulated toward dormancy release.

The targets of miR482 are several resistance (R) genes that encode a TIR (the Toll and interleukin-1 receptor) type of nucleotide binding site (NBS) -leucine-rich-repeat (LRR) receptor protein [51]. We found that the expression levels of miR482a and miR482c increased significantly during dormancy (Figure 5). As expected, we found that miR482a and miR482c target the genes belonging to the group of NBS-LRR receptors, *disease resistance protein At4g27190-like* and *putative disease resistance RPP13-like protein 1* (Table 2). In pear, miR482d was dramatically upregulated during dormancy [21,22]. As observed in previous studies, we suggest that the expression of these miRNAs may be affected by chilling [21]. In addition, a considerable amount of data from monocots indicates that miR482 targets diverse genes, including both coding and non-coding sequences. Some of these events lead to the biogenesis of additional siRNA populations known as tasiRNAs, due to ‘phasing’ activity on transcripts from so-called PHAS loci (which include PPR, NB-LRR, and MYB genes). These phased small interfering RNAs (phasiRNAs) reinforce miRNA effects on functions such as disease resistance and plant development [52].

The target of miR535m is an uncharacterized protein gene, periodic tryptophan protein 2 homolog. In *Poncirus trifoliata*, *RHOMBOID-LIKE PROTEIN 1* was identified as a target of miR535 [53]. In rice, the *SPL* genes have been identified as targets of this miRNA. Furthermore, overexpression of miR535 increased grain length, but not grain width, by repressing the expression of three target genes, *SPL7*, *SPL12,* and *SPL16* [54]. It has been shown that the miR535 and miR156 families originated from a common ancestor during evolution and together play a role in fine-tuning the expression of the *SPL* gene owing to their similarity [55]. We have observed a significantly increased expression of miR535b and miR156 during dormancy, and this same trend was previously observed in pears [21]. Both miR535b and miR156 may be related to decreased *SPL* mRNA accumulation under the conditions of this study.

The final annotated molecule, miRNA 1309, has only been found in *Pinus taeda*, and no target has so far been proposed [56]. We identified the uncharacterized gene WD repeat-containing protein 7 as a target of this miRNA. The expression of this miRNA has not yet been validated by qRT-PCR.

The miRNAs miRn1, miRn2, miRn3, miRn4, miRn5, miRn6, and miRn51 did not have homologous sequences in the latest version of plant miRBase; however, some of these molecules showed significant modulation of expression in the NGS results. Although the expression of miRn5 and miRn6 could not be validated by qRT-PCR, the same expression trend was observed by sequencing and qRT-PCR for miRn1, miRn3, miRn4, and miRn51. The expression of miRn3 and miRn51 was upregulated during dormancy (Figure 4), and we therefore propose them as target genes for the *NINJA-FAMILY PROTEIN (AFP3)* and *METAL TRANSPORTER NRAMP5-LIKE GENE*, respectively. The *NINJA-FAMILY PROTEIN (AFP3)* gene acts as a negative regulator of abscisic acid (ABA). Abscisic acid (ABA) is a phytohormone whose levels increase in response to stress in plants. *AFP3* is induced by ABA and binds to *ABI5 (ABA-INSENSITIVE5)* to suppress ABA [57]. It seems that the pathways that regulate the ABA response in dormancy play a central role, because distinct groups of genes are activated during different phases of endodormancy, including transcription factors involved in ABA signaling [34]. Specifically, during organogenesis and paradormancy, signaling pathways associated with organogenesis and ABA signaling are upregulated in sweet cherry flower buds [34].

In the case of miRn1, which we found increased toward T2, the candidate target gene was *PUTATIVE RECEPTOR PROTEIN KINASE*, a probable receptor that interacts with a ligand in the extracellular domain and triggers the protein kinase activity of the cytoplasmic domain [58]. For miRn4, we determined that the target is the *ARABINOSYLTRANSFERASE RRA3-LIKE* gene, which plays a role in the arabinosylation of extension proteins in root hair cells. Extensins are structural glycoproteins present in the cell walls, and their arabinosylation is important for root hair cell development and root hair tip growth [59]. In this study, miRn4 was downregulated toward dormancy release, which could upregulate the expression of the *RRA3-LIKE* gene and remodel the cell wall, allowing the tree to grow out of dormancy.

Although miRn2 did not show the same trend in the validation, when comparing the expression between FC and NC, it showed the same trend in qRT-PCR (Figure 5). However, no target sequence was identified for miRn2. Similarly, the other target genes proposed here have not yet been characterized. Information on the targets for all miRNAs found in our study is summarized in Supplementary Tables S6 and S7.

### 3.4. Effect of Environmental Fluctuations on the Expressions of miR156e, miR172d, miR482c, miRn1, miRn2, and miRn3 during Dormancy in Prunus avium L. var. Bing

Dormancy is one of the plant processes affected by global warming, and understanding the molecular events associated with the process and the impact caused by temperature changes is therefore important. In this regard, a comparison between FC and NC conditions showed that miR156e, miRn2, and miRn3 had the same expression pattern. This indicates that the expression of these miRNAs may be associated with normal temperature fluctuations in the field.

Other molecules, such as miR172d, miR482c, and miRn1, showed different expression patterns between FC and NC conditions. MiR172d and miRn1 also showed differences in expression between their T1-to-T2 transitions. Conversely, for miR482c, a difference was observed in the transition from T0 to T1. None of these differences led to changes in CR or flowering, as evident from the absence of change in the cold hours needed for dormancy release or the flowering process between FC and NC.

### 3.5. Inverse Correlation between miR156e and the Target Genes SPL2, 6, and 13 under Field Chilling and Non-Stop Chilling Conditions during Dormancy in Prunus avium

The regulatory crosstalk between miRNAs and their target genes has led to the miRNA node concept. Inverse correlations between target genes and miRNA expression patterns have served as preliminary evidence of this type of interaction and, consequently, post-transcriptional regulation. A well-known node involved in different floral transition processes is miR156-*SPL*. In *Arabidopsis*, the *SPL* family is comprised of 16 genes, with five of these, *AtSPL3-5*, *AtSPL9,* and *AtSPL15*, containing miRNA156 recognition sites and some functional associations with floral transition [60]. In our study, the expression of *SPL 2, 6,* and *13* genes was compared with that of miR156e under FC and NC conditions. *SPL2* was downregulated from T0 to T2 in FC, showing an inverse correlation with miRNA levels with an r value of −0.85. However, *SPL2* was upregulated from T1 to T2 in NC. *SPL6* was upregulated from T0 to T2 in FC, with an r value of 0.77, suggesting that this gene was not downregulated by miR156e. In contrast, an inverse correlation was observed in NC, with an r value of −0.91. The *SPL13* gene was down-regulated in both conditions, with r values of −0.91 and −0.76 for FC and NC conditions, respectively. This establishes an inverse correlation between the expression patterns of miR156e and *SPL13* during dormancy in sweet cherry, indicating posttranscriptional regulation. In pear, the expression of miR156 increases during dormancy [21,22], and in Populus, eight *SPL-like* genes are downregulated from paradormancy to endodormancy [61]. It has previously been suggested that downregulation of the *SPL* gene that we observed may help maintain the expression of *FLOWERING LOCUS T 2 (FT2)* and other flowering-related genes at low levels in *Populus* [61]. Although the miR156/SPL node is described as an age-dependent regulator of floral transition, these findings suggest that it could play a role in the regulation of flowering in response to temperature changes, which could be advantageous for perennial growth.

### 3.6. Future Perspectives

Unraveling the roles played by miRNA (and/or siRNA) networks in plant–environment interactions can provide an important repertoire of tools to make agriculture resilient to global climate change (GCC). In addition, both conserved and species-specific miRNAs may play important roles in plant responses to abiotic stress, and, for this reason, genetically improving plant tolerance to GCC may be possible through the application of miRNA technology. Currently, the most popular application of miRNAs and RNA interference (RNAi) is host-induced gene silencing (HIGS), which essentially focuses on the control of insect pests and viral plant diseases [62]. The use of these approaches has been restricted to genetic transformation procedures and the production of genetically modified plants. Regardless of the negative perception of the latter, genetic transformation is also hampered by the dearth of reproducible transformation protocols for many crop species, including Prunus spp. such as sweet cherry [63].

### 3.7. Conclusions

This study involved a general (NGS-based) as well as specific (qRT-PCR validation) approach to study miRNAs, a set of regulatory non-coding RNAs, during sweet cherry bud dormancy. We observed changes in miRNA expression from paradormancy to dormancy release, which indicates their potential role in dormancy progression.

We observed the opposite regulation of miR156e and miR172d expression, which indicates their participation in the sweet cherry dormancy progression. These findings are further supported by the observation of crosstalk between miR156e and the proposed target gene *PavSPL6*. According to these findings, the previously described miRNA nodes, namely miR156/*SPL*, miR172/*AP2,* and miR166/*ATBH15*, may also participate during this process.

In this study, relevant activities linked to dormancy progression were inferred through in silico modeling. During dormancy progression, regulation of stress-related genes such as *NBS-LRR* (biotic) and ABA metabolism (abiotic) takes place. In addition, our results also suggest active secondary sRNA generation (tasi and phasiRNAs) from some miR482 family members (miR482c and miR482a). At the other extreme, that is, dormancy release, increased miRNAs appear to regulate cell metabolism (*ARF*) and calcium metabolism (*ACA10*), and, consequently, cell development (*PROFILIN*, *RRA3-LIKE*, *SPL*)-associated genes.

An interesting observation was that some miRNAs and gene expression profiles did not show a correlation between field chilling (FC) and non-stop chilling (NC). Multiple mathematical models have been developed to quantify cold accumulation based on mathematical functions, mainly registered temperatures. The major problems faced by these models is that do not have any “biological” or plant in situ parameter incorporated. So far, under FC conditions, unknown factors could be affecting dormancy progression in a different way, as in NC conditions, which could explain the differences observed in this study.

This study presents some primary findings regarding dormancy in the Bing sweet cherry variety. Further validation of these findings, including through comparative studies using contrasting CR varieties, will be the next step in the understanding of the regulatory networks involved in sweet cherry dormancy, confirming the miRNA involvement described here and revealing new candidate molecules that may be useful for the development of miRNA-based strategies. Our results therefore represent the first step in the development of new tools to improve sweet cherry tree fitness and ensure fruit production in an environmentally friendly manner in the context of GCC.

## 4. Materials and Methods

### 4.1. Plant Material

Adult (8–10 years old) sweet cherry *Prunus avium* L. var. Bing trees were used. The plants were part of the collection included in the Sweet Cherry Breeding Program located at the Rayentué Experimental Station, INIA-Chile, O’Higgins Region (34°19′17″ S; 70°50′4.2″ W). Samples corresponding to floral buds and branches bearing buds were obtained from this orchard.

### 4.2. Dormancy Analyses

Dormancy stages were determined in samples generated under two experimental conditions: (a) field chilling (FC), in which samples were subjected to natural cold accumulation in the field, and (b) non-stop chilling (NC), in which samples were obtained from flower buds isolated from branches subjected to continuous chilling under cold storage.

### 4.3. Field Chilling (FC) Experiments and Temperature Data Collection

Floral buds were collected from 15 Bing trees located in the INIA-Rayentué experimental field, Rengo, in the O’Higgins region of Chile (34°19′17″ S; 70°50′4.2″ W), from autumn (April) to early spring (September).

Chilling hours under field conditions were calculated using the chilling hours model, that is, hours < 7.2 °C [64]. Temperature data were collected from the Red Agroclimática Nacional INIA-FDF (Fundación para el Desarrollo Frutícola Station, located in the same experimental field).

### 4.4. Chilling Requirement Determination under Forcing Conditions

Branches bearing four to six floral bud clusters were randomly collected. Branches were incubated in water pots and placed under forcing conditions at 25 °C under a 16/8 h day/night photoperiod. After a week, the phenological status of floral buds was scored, and the chilling requirement was considered fulfilled when at least 50% of the buds burst and began to show sepals in the BBCH 53 stage after 14 days of forcing conditions [14].

### 4.5. Non-Stop Chilling (NC) Experiments

To avoid temperature variations caused by field conditions, continuous cold accumulation of tree sticks was conducted through non-stop chilling storage in a cold chamber. Branches bearing four to six floral bud clusters were collected from three different trees in the same orchard used for the FC experiments at the beginning of autumn. Branches were transferred to the laboratory, disinfected, divided into groups of five to six branches, and wrapped using fungicide solution-moistened papers. The branches were kept at 4–6 °C in the dark for progressive chilling accumulation. After reaching a specific chilling time point, each stored group was split into branches for chilling requirement determination and phenological evaluation in the greenhouse, as described previously, and branches from the floral buds were sampled and frozen for molecular analysis.

### 4.6. RNA Isolation

Floral buds from FC and NC experiments were subjected to RNA isolation. Buds were cut off from branches immediately before the stick heat activation process, immediately frozen in liquid nitrogen, and stored at −80 °C. Total RNA was extracted from flower buds using the Plant RNA Reagent (Invitrogen) according to the manufacturer’s instructions. RNA was quantified using the Qubit system (Invitrogen), and RNA integrity was verified on a 1.5% MOPS denaturing gel and by the Fragment Analyzer system (DNF-472; Advanced Analytical Technologies), using the criterion of RNA quality number (RQN) > 8.0 for the sRNA sequencing libraries and RQN > 7.0 for qRT-PCR analysis.

### 4.7. Genome-Wide Identification of Cherry miRNAs and Their Expression

Library assembly, processing, and Illumina sequencing were performed by Macrogen (Republic of Korea). NGS raw data were trimmed and filtered using the REAPR program [65], and repeated sequences and other classes of RNAs were removed using the RepBase [66] and Rfam databases [67], respectively. The Bowtie tool was used to map the sequences against the *Prunus avium* ‘Karina’ (2n = 2x = 16) genome, previously drafted in our laboratory [68]. MiR-PREFeR was used for miRNA prediction [69], SAMTools was used to identify candidate regions in the genome, and the RNAfold web server was used to predict secondary structures. miRNA annotations were obtained from the miRBase database (version 22), considering the best scores (Supplementary Tables S2 and S3). The structures of the precursors of new miRNAs were determined using the Mfold server configured with default parameters (Supplementary Table S4). Normalization and hierarchical clustering were performed using DeSeq2-Bioconductor for the identification of differentially expressed genes, which was based on determining the number of reads in each library of each mature miRNA. These data were transformed into normalized reads as transcript per million values (TPM, Supplementary Tables S2–S4) using the following formula: normalized expression = miRNA count/(total count of reads × 1,000,000).

### 4.8. Prediction of Target Genes

The target genes for the identified miRNAs were deduced using psRNATarget (https://www.zhaolab.org/psRNATarget/ (accessed on 12 October 2021), using the *Prunus mume* genome draft as a reference [32]. The main parameters used for target gene selection were the unpaired energy (UPE; i.e., the energy required to open the secondary structure formed by the miRNA and mRNA) applying UPE < 20.0 and expectation < 3.0, respectively, as thresholds (Supplementary Tables S5–S7). Furthermore, the targets’ function was used as an additional criterion of relevance and revealed using the Gene Ontology (GO) tool (http://geneontology.org/ (accessed on 12 October 2021)).

### 4.9. Experimental Determination of microRNAs and Target Genes

One microgram of total RNA was incubated with *DNAse* I (Thermo Fisher Scientific (Waltham, MA, USA)) and checked for genomic DNA contamination by PCR amplification of the second intron of the *PavDAM5* gene (Supplementary Table S8) [14]. cDNA-miRNAs were obtained from these DNA-free RNA extracts using stem-loop-based qRT-PCR [70]. The structural sequence of the primer required for the stem loop was based on the sequence 5’-GTCGTATCCAGTGCAGGGTCCGAGGTATTCGCACTGGATACGACNNNNNN-3’, in which the positions marked by N are miRNA specific. The 3′-end primer for each miRNA amplification reaction had a 6-base overhang for specific interactions between the primer and the target miRNA (Supplementary Table S8). miRNA-cDNA of each selected miRNA was synthesized with the Superscript First-Strand kit (Invitrogen), according to the manufacturer’s protocol, in which 2 *p*mole of each primer for stem-loop qRT-PCR (Supplementary Table S8), 1 μL of 10 mM dNTP mix, and nuclease-free water to a final volume of 12 μL were used. For target gene amplification, first-strand cDNA synthesis was performed using oligo-dTs in the reverse transcription step.

Expression patterns were assessed using qRT-PCR. Briefly, each reaction was run in triplicate with 1 µL of cDNA in a 20-µL final volume using 0.6–0.8 µM of the corresponding primers (Supplementary Table S8) and 1X Eva Green master mix (Biotium). The analysis was performed using a G8830A AriaMx Real-time PCR System (Agilent Technologies) (Supplementary Table S9). The 2-ΔΔCT method was used for relative quantification of miRNA and normalized using sRNA obtained from the sRNA sequencing of cherry flower buds, noted as precursor_393, and using the *β-actin* transcript, for expression of target genes [71].

## Figures and Tables

**Table 1:** Observed budburst from branch samples of Bing variety with different CRs obtained from FC and NC conditions.

FC ^1^		NC ^2^	
Chilling Hours	Observed Bud Burst	Chilling Hours	Observed Bud Burst
0 (T0)	0%	0 (T0)	0%
254	0%	218	0%
356	0%	386	0%
545	0%	530	0%
693	0%	698	0%
853 (T1)	14%	890 (T1)	36%
909 (T2)	58%	914 (T2)	58%
989	83%	1250	87%

^1^ Field chilling. ^2^ Non-stop chilling.

**Table 2:** Summary of notation and characterization of *Prunus avium* miRNAs and their targets genes.

Name	Best Match miRBase	Mature Sequence (5’-3’)	miRNA Levels	Target(s) (psRNAtarget)	GO-Molecular Function	GO-Biological Process	Function
miR156e	ppe-miR156e	UGACAGAAGAGAGUGAGCAC	Increased	Squamosa promoter-binding-like protein	DNA binding; DNA binding transcription factor activity; metal ion binding	Defense response to bacterium; regulation of gene expression; regulation of transcription, DNA-templated; anther development (SPL13)	Trans-acting factor that binds specifically to the consensus nucleotide sequence 5’-TNCGTACAA-3’.
miR166c	ppe-miR166c	UCGGACCAGGCUUCAUUCCCC	Decreased	Homeobox-leucine zipper protein ATHB-15	DNA binding; lipid binding	Cell differentiation; regulation of transcription, DNA-templated	Probable transcription factor involved in the regulation of meristem development to promote lateral organ formation. May regulate procambial and vascular tissue formation or maintenance and vascular development in inflorescence stems.
miR172d	ppe-miR172d	GGAAUCUUGAUGAUGCUGCAG	Decreased	AP2-like ethylene-responsive transcription factor	DNA binding; DNA binding transcription factor activity	Ethylene-activated signaling pathway; multicellular organism development; transcription, DNA templated	Probably acts as a transcriptional activator. Binds to the GCC-box pathogenesis-related promoter element. May be involved in the regulation of gene expression by stress factors and by components of stress signal transduction pathways (by similarity). May negatively regulate the transition to flowering time and confers flowering time delay.
miR391	mdm-miR391	UACGCAGGAGAGAUGGCGCUG	Increased	Profilin	Actin monomer binding	Actin polymerization or depolymerization; inflorescence development; lateral root development; leaf development; sequestering of actin monomers; unidimensional cell growth	Binds to actin and affects the structure of the cytoskeleton. At high concentrations, profilin prevents the polymerization of actin, whereas it enhances it at low concentrations. By binding to PIP2, it inhibits the formation of IP3 and DG (by similarity).
miR482a	ppe-miR482a-3p	UUUCCGAAACCUCCCAUUCCAA	unchanged	Disease resistance protein At4g27190-like	ADP binding; ATP binding	Defense response; signal transduction	Disease resistance protein
miR482c	ppe-miR482c-3p	UUGCCAACCCCGCCCAUUCCAA	Increased	Putative disease resistance RPP13-like protein 1	ATP binding	Plant-type hypersensitive responser; signal transduction	Potential disease resistance protein
miR535b	ppe-miR535d	UUGACGACGAGAGAGAGCACG	Increased	Periodic tryptophan protein 2 homolog	RNA binding; snoRNA binding	Maturation of SSU-rRNA from tricistronic rRNA transcript (SSU-rRNA, 5.8S rRNA, LSU-rRNA); ribosomal small subunit assembly; rRNA processing	Not in plants
miR1309	pta-miR1309	UUGAUGGACCAUUUGAAUGAA	not validated	WD repeat-containing protein 7	Hematopoietic progenitor cell differentiation	/	Not present in plants
miRn51	/	UUUGGCGCGUUGCUGUGGAUU	Increased	Metal transporter Nramp5-like	/	Metal ion transmembrane transporter activity	/
miRn1	/	CAUAGGAUGCUUAGGAAACUU	Increased	Putative receptor protein kinase	ATP binding; protein serine/threonine kinase activity	Recognition of pollen, self-incompatibility	Probable receptor. Interaction with a ligand in the extracellular domain triggers the protein kinase activity of the cytoplasmic domain.
miRn2	/	UAGUCAAUUAAUGAGGAUUAGU	unchanged	n.a	/	/	/
miRn3	/	UUUUCUGAAGCAUUUGGCAUC	Decreased	Ninja-family protein [AFP3]	/	Signal transduction; protein binding	Acts as a negative regulator of abscisic acid (ABA) response and stress responses.
miRn4	/	UUCUUGGAGGCAUGAAGCACC	Increased	Arabinosyltransferase RRA3-like	Transferase, transfering glycosyl groups	Cell wall biogenesis; cell wall organization; root hair cell development	Plays a role in the arabinosylation of cell wall components. Involved in the arabinosylation of extensin proteins in root hair cells. Extensins are structural glycoproteins present in cell walls, and their arabinosylation is important for root hair cell development and root hair tip growth.
miRn5	/	UGGCAUCGAGGACGAACAGCU	not validated	psbP domain-containing protein 7, chloroplastic	Calcium ion binding	Photosynthesis	/
miRn6	/	UUACAAAGUAUCUUAUGGGUCU	not validated	Pentatricopeptide repeat-containing protein At1g32415, mitochondrial	Endonuclease activity; RNA binding	RNA modification	/

## Data Availability

Not applicable.

## Supplementary Materials

The following supporting information can be downloaded at: https://www.mdpi.com/article/10.3390/plants11182396/s1, Figure S1: PCR product sequencing of miRNAs; Figure S2: miRNAs and nodes deduced for sweet cherry dormancy; Table S1: Statistics sequencing of sRNAs of sweet cherry flower buds during dormancy; Table S2: Annotation, alignment, and expression analyses for highly conserved miRNAs and their precursors; Table S3: Annotation, alignment, and expression analyses for conserved miRNAs and their precursors; Table S4: Annotation, alignment, and expression analyses for unknown miRNAs and their precursors; Table S5: Search of highly conserved and conserved miRNA targets; Table S6: Search of conserved miRNA targets; Table S7: Search of new miRNA targets; Table S8: Description of primers; Table S9: Cycle quantification value (Cq) of miRNAs and target genes determined by qRT-PCR.
