# Supplementary material for: Small RNA Differential Expression Analysis Reveals miRNAs Involved in Dormancy Progression in Sweet Cherry Floral Buds
Source: Plants (Basel). 2022 Sep 14;11(18):2396. doi: 10.3390/plants11182396 (PMC9500734; doi:10.3390/plants11182396)
Supplement: Supplementary file 1 [file plants-11-02396-s001.zip › Supplementary Figure S2.pdf]

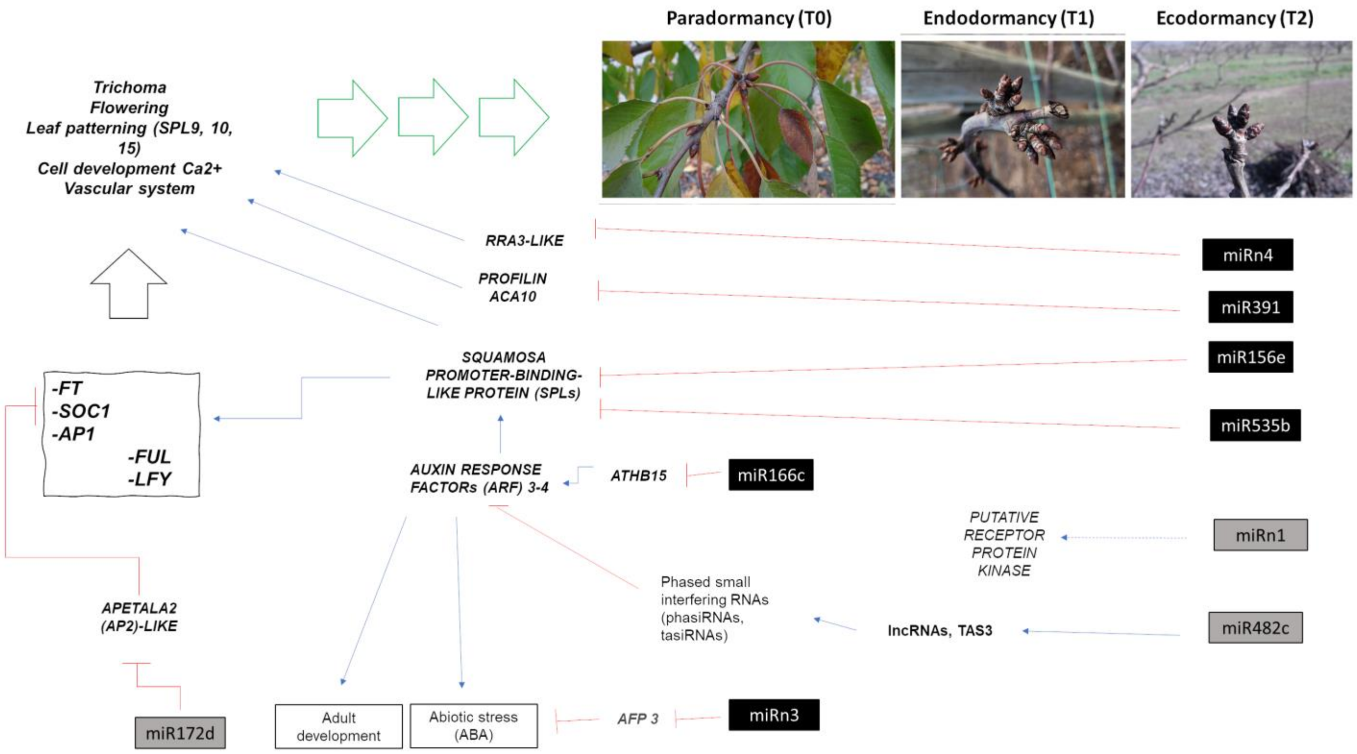

Figure S2. MicroRNAs and nodes deduced for sweet cherry dormancy. Several miRNA nodes could guide various aspects of dormancy; prominent roles of the molecules found in this work are proposed for dormancy progression and release. Metabolic and physiological events taking place during the process include transition from juvenile to adult plant-development and stress related processes. Increased presence of miRNA molecules found during dormancy (black boxes) and their possible gene targets are shown either for an inhibitory (red line) or activating (blue lines) conditions. In general terms, increased levels of miR166 (T0) could indicate *HOMEOBOX-LEUCINE ZIPPER PROTEIN* (*ATHB15*) mRNA regulation, which could lead to *ATHB15*-mediated responses such as maintenance of the vascular system and of genes associated to abscisic acid (ABA) response. At dormancy release, increased levels of miR391, which targets *PROFILIN* and *ACA-10* genes, could lead to active cell growing (actin binding and calcium transport) events associated to plant development, immunity, and response to environmental stimulus. Increased miR391 could also be silencing *AUXIN RESPONSE FACTORS* (*ARF*s). In addition, miR482, significantly increased during dormancy, could be leading to *NUCLEOTIDE BINDING SITE-LEUCINE-RICH REPEAT* (*NBS-LRR*) and *tasiR-ARF* (*TAS*) regulation, representing an important phased small interfering RNAs (phasiRNAs, tasiRNAs) biogenesis reinforcing functions such as disease resistance and plant development. Black boxes indicate molecules with consistent accumulation patterns between NGS and qRT-PCR approaches (Pearson's correlation coefficient ≈ 1 between data sets). Grey boxes represent molecules with uneven trend between methods.
